# Stat3 Inhibitor Stattic Exhibits Potent Antitumor Activity and Induces Chemo- and Radio-Sensitivity in Nasopharyngeal Carcinoma

**DOI:** 10.1371/journal.pone.0054565

**Published:** 2013-01-29

**Authors:** Yunbao Pan, Fuling Zhou, Ronghua Zhang, Francois X. Claret

**Affiliations:** 1 Department of Systems Biology, The University of Texas MD Anderson Cancer Center, Houston, Texas, United States of America; 2 Department of Pathophysiology, Zhongshan School of Medicine, Sun Yat-Sen University, Guangzhou, Guangdong, People’s Republic of China; 3 Department of Clinical Hematology, the Affiliated No. 2 Hospital, Xi’an JiaoTong University, Xi’an, Shanxi, People’s Republic of China; 4 Experimental Therapeutic Academic Program and Cancer Biology Program, The University of Texas Graduate School of Biomedical Sciences at Houston, Houston, Texas, United States of America; The Chinese University of Hong Kong, Hong Kong

## Abstract

Nasopharyngeal carcinoma (NPC) is an Epstein-Barr virus-associated malignancy most common in East Asia, Africa and Alaska. Radiotherapy and cisplatin-based chemotherapy are the main treatment options. Unfortunately, disease response to concurrent chemoradiotherapy varies among patients with NPC, and many cases are resistant to cisplatin and radiotherapy. Signal transducer and activator of transcription 3 (Stat3) has been implicated in the development and progression of various solid tumors. In this study, we assessed the activation and expression of Stat3 in NPC cells. We found that Stat3 was activated and could be blocked by the small molecule inhibitor Stattic. The inhibition of Stat3 in NPC cells by Stattic decreased the expression of cyclin D1 in a dose- and time-dependent manner. Thus, Stattic was used to target Stat3 in NPC cell lines. We found that Stattic could inhibit cell viability and proliferation in NPC cells and significantly induced apoptosis. Additionally, Stat3 transfection attenuated, whereas Stat3 knockdown enhanced, the effects of Stattic upon cell viability inhibition and apoptosis induction. Furthermore, Stattic sensitized NPC cells to cisplatin and ionizing radiation (IR) by preventing cell proliferation and inducing apoptosis. Taken together, Stattic inhibit Stat3 and display antitumor effect in NPC, and enhanced chemosensitivity and radiosensitivity in NPC. Therefore, our findings provide the base for more rational approaches to treat NPC in the clinic.

## Introduction

Nasopharyngeal carcinoma (NPC) arises from the epithelial lining of the nasopharynx [1] and is one of the most poorly understood types of cancer. NPC has a remarkable ethnic and geographic distribution, with a high prevalence in southern China, Southeast Asia, Northern Africa, Greenland and Inuits of Alaska [2]. The annual incidence peaks at 50 cases per 100,000 persons in endemic regions, but it is rare in the Western world (1 per 100,000 persons) [3]. Epstein-Barr virus (EBV) infection, environmental factors, and genetic susceptibility are associated with NPC [1], [2], [4].

Cisplatin chemotherapy and radiotherapy are the main treatments for NPC [5], [6]. Unfortunately, many NPC patients do not benefit much from concurrent chemoradiotherapy; 30% to 40% of patients develop distant metastases within 4 years [7], and once metastasis occurs, the prognosis is very poor. Genetic alterations have been reported in NPC, and our recent findings showed that Jab1/CSN5 is overexpressed and negatively regulates p27 in NPC [8] and contribute to radiotherapy and chemotherapy resistance [9], [10]. There is a critical need to develop more effective treatments for NPC.

Signal transducer and activator of transcription 3 (STAT3) is a member of a family of latent cytosolic transcription factors whose activation is contingent on the phosphorylation of a conserved tyrosine residue (Y705) by upstream kinases such as Janus kinase 2 (JAK2) [11]. This event promotes the dimerization of STAT3 monomers via their Src homology-2 (SH2) domains, rendering them in a transcriptionally active conformation [12]. Persistent activation of the JAK2/STAT3 signaling pathway has been documented in a wide range of human solid and blood cancers and is commonly associated with worse prognoses [13], [14]. Among the Tumor-promoting activities ascribed to persistent STAT3 signaling are those involved with cell proliferation, metastasis, angiogenesis, host immune evasion, and resistance to apoptosis [15], [16]. STAT3 is constitutively activated and expressed in the nucleus in NPC cells [17] and it has been reported that stat3 activation in NPC is induced by EBV encoded LMP1 [18]. Recently, it has been reported that STAT3 activation contributes directly to the invasiveness of nasopharyngeal cancer cells [19]. Although STAT3 serves critical and necessary roles in early embryogenesis, its presence in the majority of normal adult cell types is largely dispensable [20], making it an attractive target for cancer therapy.

Different approaches have been developed to effectively inhibit STAT3 [21]. In silico screenings to identify candidate non-peptidic small molecules that inhibit STAT3 by binding directly to its Src homology 2 (SH2) domain led to a whole new class of inhibitors [22], [23]. Of these, the commercially available inhibitor Stattic has been shown to selectively inhibit the function of the STAT3 SH2 domain regardless of STAT3 phosphorylation status [24]. Stattic selectively inhibits activation, dimerization, and nuclear translocation of STAT3, resulting in an increase in apoptosis rates of STAT3-dependent cancer cells [24], [25]. Despite an abundance of work focused on the inhibition of Stat3 activation, the anti-tumor effects on NPC have not yet been reported. The purpose of this work is to provide an initial assessment of the potential therapeutic utility of STAT3 inhibition by Stattic in NPC.

Our findings indicate that Stattic, through inhibition of STAT3 activation, reduces the growth and increases the apoptosis of NPC and sensitize NPC to cisplatin and IR. This work identifies Stattic as a potential targeted therapy that sensitize cells prior to conventional chemotherapy and radiotherapy, thus providing more effective treatment for NPC patients.

## Materials and Methods

### Reagents

Cell culture medium was from Mediatech Inc. (Manassas, VA, USA) and fetal bovine serum (FBS) from Gibco (Grand Island, NY, USA). The antibodies used were PARP (BD Biosciences PharMingen, San Diego, CA, USA), caspase-3, total Stat3, p-Stat3, and cyclin D1 (Cell Signaling Technology, Beverly, MA, USA), β-actin and FLAG-tag (Sigma-Aldrich, St. Louis, MO, USA). The caspase-3 colorimetric assay kit was from GenScript (Piscataway, NJ, USA). Lipofectamine Plus reagent and Oligofectamine reagent were from Invitrogen (Carlsbad, CA, USA), Western Lightning Chemiluminescence Plus reagent was from Thermo Scientific Pierce (Rockford, IL, USA), and the Cell Proliferation Kit was from Roche (Indianapolis, IN, USA). IL-6 was obtained from Invitrogen and used at 40 ng/mL. Stattic inhibitor was purchased from Sigma (St. Louis, MO, USA).

### Cell Cultures

EBV-negative NPC cell lines CNE1, CNE2, HONE1 and EBV-positive NPC cell line C666-1 were cultured in RPMI medium containing 10% FBS and penicillin-streptomycin sulfate as described previously [8]. HOK16B (normal keratinocyte cells) were cultured in keratinocyte-SFM medium containing 30 mg/ml bovine pituitary extract, 0.2 ng/ml epidermal growth factor (EGF), 5% FBS, and penicillin-streptomycin sulfate as described previously [8], and 8 hours before harvesting protein for western blotting, the medium was changed into the same medium that cultured NPC cells. All cell lines were incubated at 37°C in an atmosphere of 5% CO_2_.

### Plasmid and Small Interfering RNA (siRNA) Transfection

The Flag-Stat3-pCDNA3.1 and vector control plasmids has been described previously [26]. Cells were transfected using the Lipofectamine Plus reagent as described previously [9]. The negative control (siControl) gene products and siRNA targeting the human Stat3 were purchased from Open Biosystems. Transient transfections of NPC cells were performed using the Oligofectamine (Invitrogen) protocol and concentrations of siRNAs at 5 nmol in RPMI with 10% FBS and no penicillin-streptomycin.

### Cell Viability Assay

The 3-(4,5-dimethylthiazol-2-yl)-2,5-diphenyltetrazolium bromide (MTT) assay was used to evaluate cell viability as described previously [9]. Cell were γ-irradiated using a JL Shepherd and Associates (CA) Mark I-30 ^137^Cs irradiator at MD Anderson Cancer Center. Briefly, the NPC cells were seeded into 96-well plates in RPMI-1640 medium with 10% FBS. After the indicated treatment and incubation period, the MTT labeling reagent was added, and the spectrophotometric absorbance of the samples was read using a microplate (ELISA) reader at 570 nm. The data were analyzed using GraphPad Prism 4 (GraphPad Software, La Jolla, CA, USA).

### Colony Formation Assay

We performed the colony formation assay as previously described [9]. Generally, NPC cells (400 cells/well) were plated in six-well plates, and the next day cells were exposed to the indicated treatment. After 10 days, the cells were fixed, stained with 0.1% crystal violet, and scored by counting colonies under an inverted microscope, using the standard definition that a colony consists of 50 or more cells.

### Hoechst 33342 Staining

To detect apoptosis, we performed nuclear staining as described previously [9] using 10 mg/ml Hoechst 33342, and cells were analyzed with a fluorescence microscope (magnification ×200 for nuclear analysis and ×100 for morphologic analysis). Apoptotic cells were identified by morphology and by condensation and fragmentation of their nuclei. The percentage of apoptotic cells was calculated as the ratio of apoptotic cells to total cells counted, multiplied by 100. Three independent experiments were conducted, and at least 300 cells were counted for each experiment.

### Caspase-3 Colorimetric Assay

The levels of an apoptosis marker, caspase-3 (active form), were measured in cell lysates using a colorimetric assay kit (cat. no. L00289; GenScript), with the assay performed according to the manufacturer’s instructions. NPC cells were treated with Stattic for 48 h prior to the assay. Cell extracts were incubated with caspase-3 substrate at 37°C for 4 h. The reaction was measured at 405 nm in a microplate reader. We subtracted background readings for cell lysates and buffers from the readings of both Stattic-induced and control samples before calculating the relative change increase in caspase-3 activity in the Stattic-induced samples compared with the control. To measure increases in caspase-3 activities in Stattic-treated samples, we normalized increases to the caspase-3 activity of the untreated sample, which was set to 1.0 fold.

### Flow Cytometry Analysis of the Cell Cycle

Propidium iodide (PI) staining was performed as described previously [9]. Briefly, the treated cells were fixed overnight, washed in cold phosphate-buffered saline (PBS), labeled with PI, and analyzed immediately after staining using a FACScan flow cytometer (BD Biosciences) and WinMDI29 software.

### Cell Extracts and Immunoblotting

Cells in the log phase of growth were collected, washed twice in cold PBS, and lysed as described previously [8]. Proteins in the total cell lysates were separated by 10% sodium dodecyl sulfate–polyacrylamide gel electrophoresis (SDS-PAGE), transferred to nitrocellulose membranes, and probed with anti-T-Stat3, anti-p-Stat3, anti-PARP, anti-caspase-3, and anti-cyclin D1. -Actin was used as the internal positive control for all immunoblots. Immunoreactive bands were detected using HRP-conjugated secondary antibodies with the Western Lightning Chemiluminescence Plus reagent. The protein levels were quantified using ImageJ software (National Institute of Mental Health, Bethesda, MD, USA; http://rsb.info.nih.gov/ij). Activities of PARP and caspase-3 were measured as the proportion of cleavage band intensity to the total bands and calculated as follows: % PARP or caspase-3 = 100% × Tc/Tt, where Tc is the intensity value of the cleavage bands and Tt is the intensity value of total bands.

### Statistical Analysis

Statistical analysis for the results was performed using Student’s *t* test for only two groups or using one-way analysis of variance when there were more than two groups. Differences between groups were considered statistically significant when *P*<0.05. All computations were performed with SPSS 19.0 (SPSS, Chicago, IL, USA).

## Results

### IL-6/Stat3 Signaling in NPC

**Figure 1:**
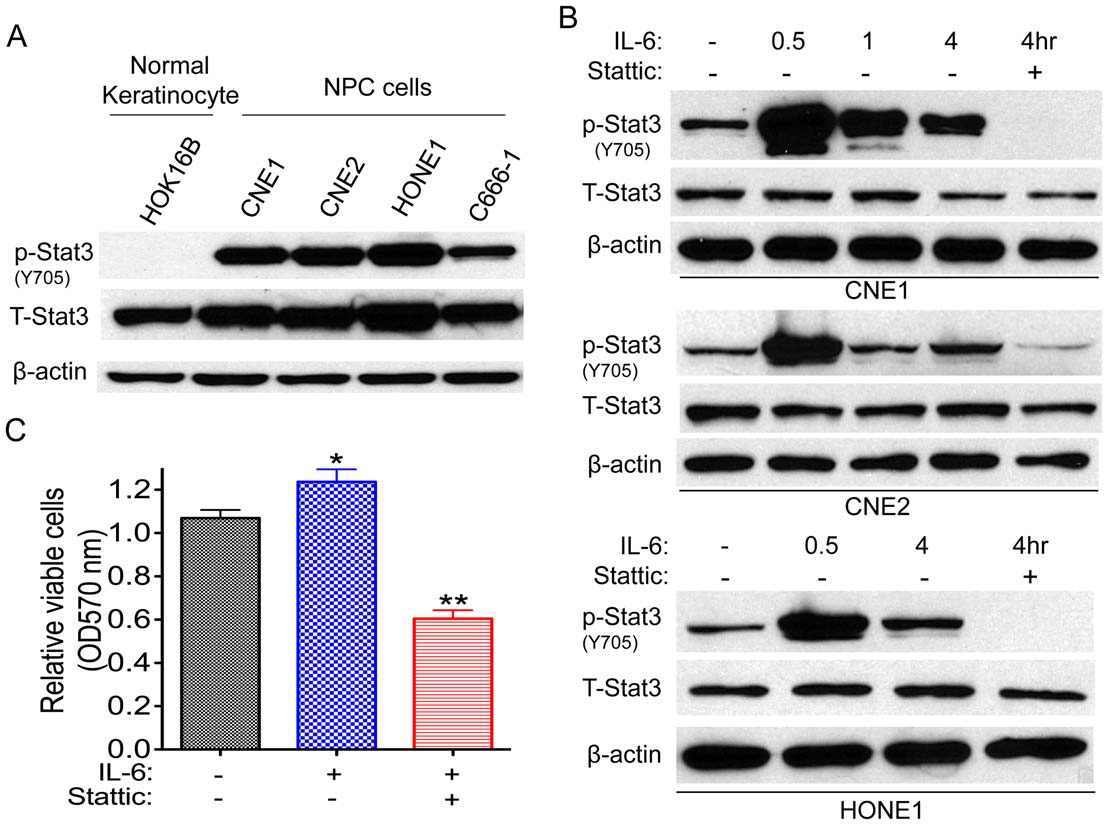
IL-6/Stat3 signaling in NPC cells. (A) Whole-cell lysates were prepared from the cells as indicated. -Actin was used as a control for protein loading and integrity. The relative phosphorylated Stat3 (p-Stat3) and total Stat3 (T-Stat3) expression intensity from 5 samples is shown. (B) Treatment with IL-6 (40 ng/mL) activated Stat3, [P-Stat3(Y705)] and such activation was inhibited by the addition of Stattic (20 µM ) in NPC cells. (C). IL-6 (40 ng/mL) promoted CNE1 cell growth, and the growth was inhibited by the addition of Stattic (4 µM ). Data are means ± s.d. for three independent experiments, *P<0.05, **P<0.01. DMSO were used as control in “−” groups.

We first examined the Stat3 expression in NPC cells. Immunoblotting showed strong total Stat3 and phosphorylated Stat3 expressions in NPC cells but not in normal keratinocyte cells, where weak Stat3 expression was detected (Fig. 1A), indicating that Stat3 is overexpressed in NPC. We further investigated whether an upstream activator of Stat3, the cytokine IL-6, could be driving increased Stat3 expression in NPC. Treatment of CNE1 cells with IL-6 for 30 min increased phosphorylation of Stat3 on tyrosine 705 (Y705) (Fig. 1B) within the short time of 30 min, as well as at 1 h and 4 h, and this phosphorylation was partially blocked by the addition of the Stat3 inhibitor Stattic. The same trends were observed in the NPC cell lines CNE2 and HONE1 (Fig. 1B). IL-6 also resulted in increased cell viability in CNE1 cells by approximately 24%, a result that is also supported by the findings of Tu et al. in Saos-2 cells [27]; however, Stattic significantly reduced cell viability by 50% as measured by the MTT assay (Fig. 1C).

### Stattic Action is Dose and Time Dependent

**Figure 2:**
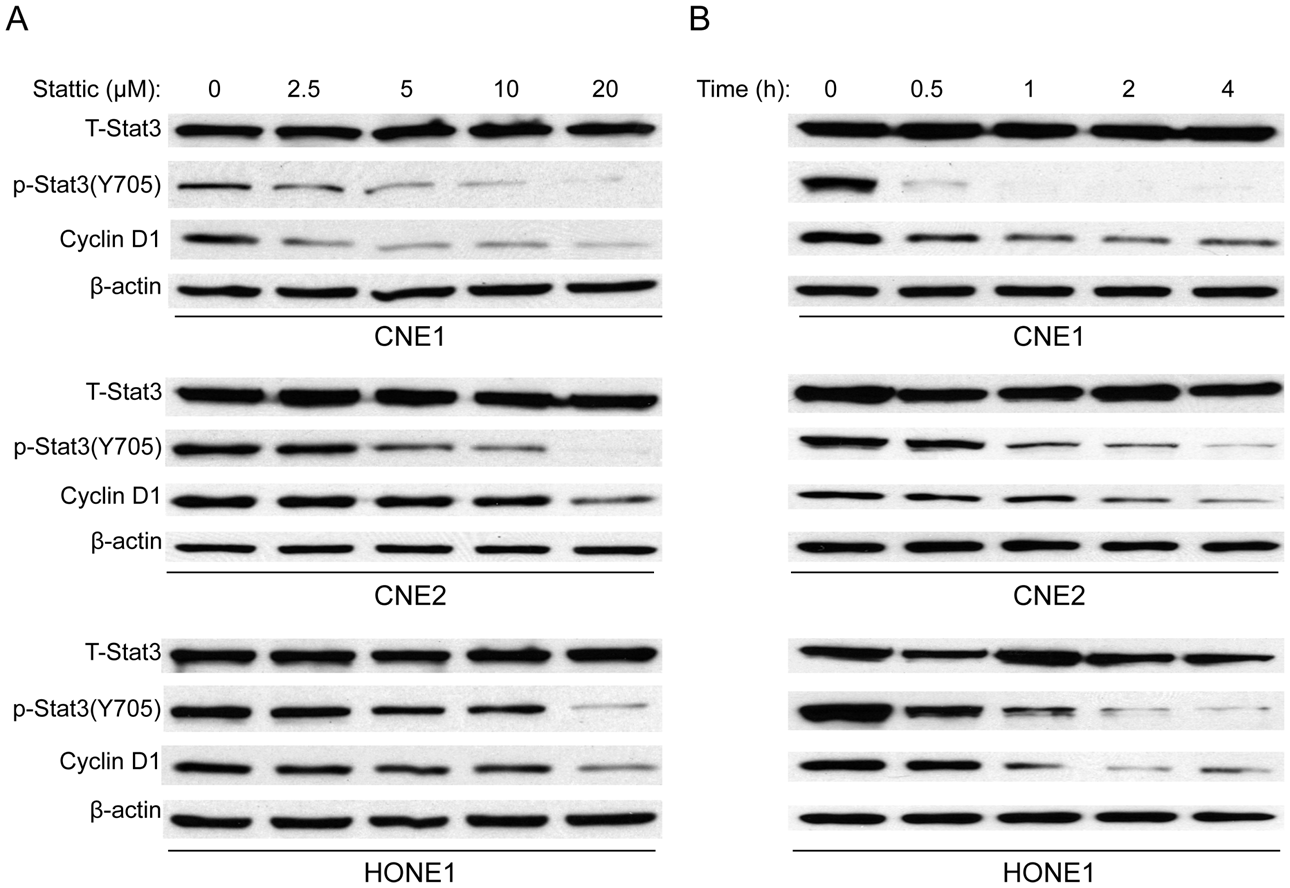
Stattic inhibits Stat3 activation in a dose- and time-dependent manner in NPC cells. NPC cell lines were treated with and without Stattic at the specified concentrations for 2 h (A) or exposed to 20 µM Stattic for different time points (B). Expression of T-Stat3, p-Stat3, cyclin D1, and β-actin were examined by western blot. DMSO were used as control in “0” groups.

As discussed above, Stattic inhibition of IL-6 induced Stat3 phosphorylation. To further determine the effect of Stattic on Stat3 activation in NPC, we exposed three NPC cell lines to various concentrations of Stattic. As shown in Fig. 2A and B, Stattic inhibited the Stat3 activation in a dose- and time-dependent manner. As was the case with Stat3 activation, cyclin D1, a target gene of Stat3, was likewise downregulated after treatment with Stattic. These data suggest that Stattic inhibits Stat3 activation in NPC.

### Stattic Inhibited Cell Viability and Arrested Cell Cycle in NPC

**Figure 3:**
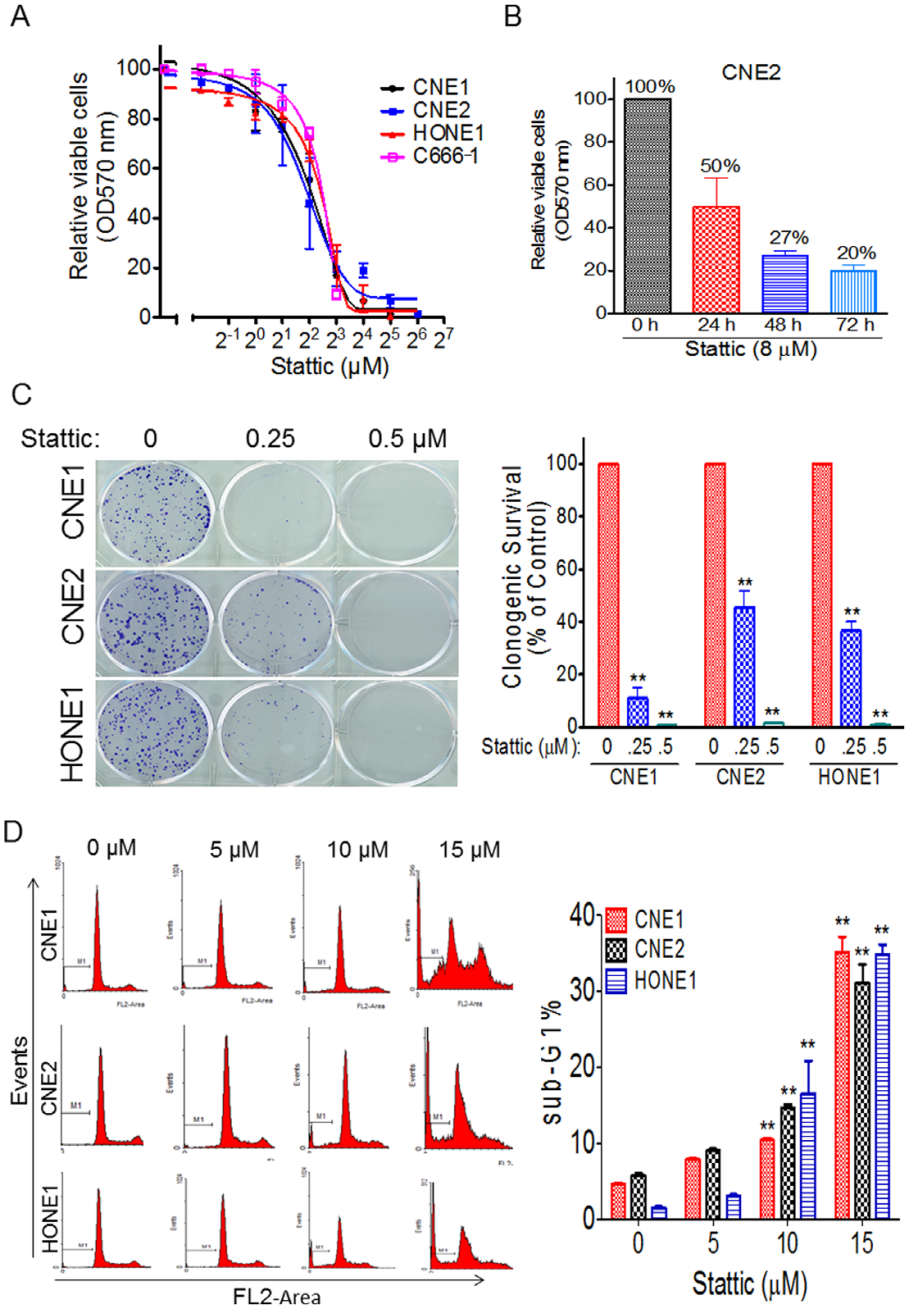
Stattic inhibits cell viability and induces sub-G1 arrest in NPC cells. (A) Cell viability assay. CNE1, CNE2, HONE1 and C666-1 cells were treated with various doses of Stattic for 48 h. Cell viability was measured by the MTT assay. (B) Stattic inhibits the cell viability of NPC cells in a time-dependent manner. CNE2 cells were treated with 8 µM Stattic for the indicated times, and cell viability was measured by the MTT assay. (C), (Left) Representative results of colony formation assays with NPC cells treated with different doses of Stattic. (Right) Quantification of the relative number of colonies is shown. (D) Measurement of apoptosis by PI staining. (Left) NPC cells were treated with the indicated doses of Stattic for 48 h, followed by PI staining as described in Materials and Methods. (Right) Quantification of PI staining. Data are means ± s.d. for three independent experiments, *P<0.05, **P<0.01. DMSO were used as control in “0” groups.

After establishing the efficacy of Stattic as a selective Stat3 inhibitor in NPC, we next examined its growth-suppressive activity in NPC. We exposed four NPC cell lines to different concentrations of Stattic. In our studies, Stattic showed growth-suppressive activity in the NPC cell lines tested in a dose- and time-dependent manner (Fig. 3A and B). We further performed a colony formation assay to test the effect of Stattic on NPC cells’ proliferation. As expected, Stattic significantly inhibited colony formation, with over 98% inhibition at 0.5 µM treatment in all three NPC cell lines tested (Fig. 3C). Consistent with the observations seen in NPC cells, flow cytometric analysis revealed that 35% of the CNE1 cells had hypodiploid (sub-G1) DNA content, reflecting apoptosis, whereas 31% of CNE2 and 34% of HONE1 had hypodiploid DNA after 15 µM Stattic treatment (Fig. 3D).

### Stattic Induced Apoptosis in NPC

**Figure 4:**
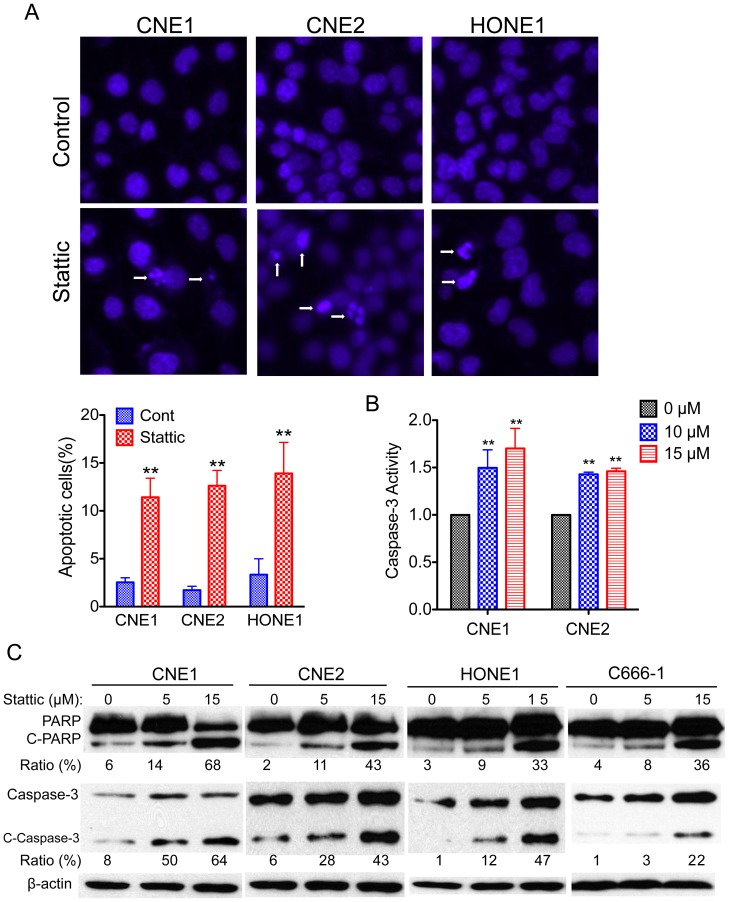
Stattic induces apoptosis in NPC cells. (A) Apoptosis was measured by Hoechst 33342 staining. (Top) NPC cells were treated with 10 µM Stattic for 48 h, nuclei were stained with Hoechst 33342, and imaging analysis was performed as described in the Materials and Methods. The white arrows indicate apoptotic cells. Original magnification, ×200. (Bottom) Quantification of the cell staining. (B) Effect of Stattic on caspase-3 activity. The cells were treated with the indicated concentrations of Stattic for 48 h. The activities were determined as described in Materials and Methods. (C) NPC cells were exposed to the indicated concentrations of Stattic for 48 h; apoptotic cells were measured by western blot analysis of cleaved PARP and cleaved caspase-3. Protein levels were quantified using ImageJ software. Data are means ± s.d. for three independent experiments, *P<0.05, **P<0.01. DMSO were used as control in “0” groups.

We next determine whether the Stattic-induced cell viability inhibition is followed by increases in apoptosis. CNE1, CNE2, and HONE1 cells were treated with Stattic for 48 h and analyzed by Hoechst 33342 staining, which detects condensed nuclei, an indicator of apoptosis. Treatment of NPC cells with Stattic resulted in a marked increase in the number of apoptotic cells, with the number of apoptotic cells being 4.6 times higher in CNE1 cells, 5.7 times higher in CNE2 cells, and 4.2 times higher in HONE1 cells (Fig. 4A). To confirm these findings with an independent assay, we measured apoptosis by the caspase-3 colorimetric assay. Forty-eight hours after 15 µM Stattic exposure, the caspase-3 activities were 1.7 times higher in CNE1 cells and 1.5 times higher in CNE2 cells compared with DMSO treated control cells (Fig. 4B). Because cleavage of poly (ADP-ribose) polymerase (PARP) and caspase-3 activation are hallmarks of the initiation of apoptosis [28], [29], we further examined the influence of Stattic on NPC cells. As expected, Stattic induced PARP and caspase-3 cleavage in a dose-dependent manner (Fig. 4C).

### Stat3 Expression was Associated with Stattic Efficacy

**Figure 5:**
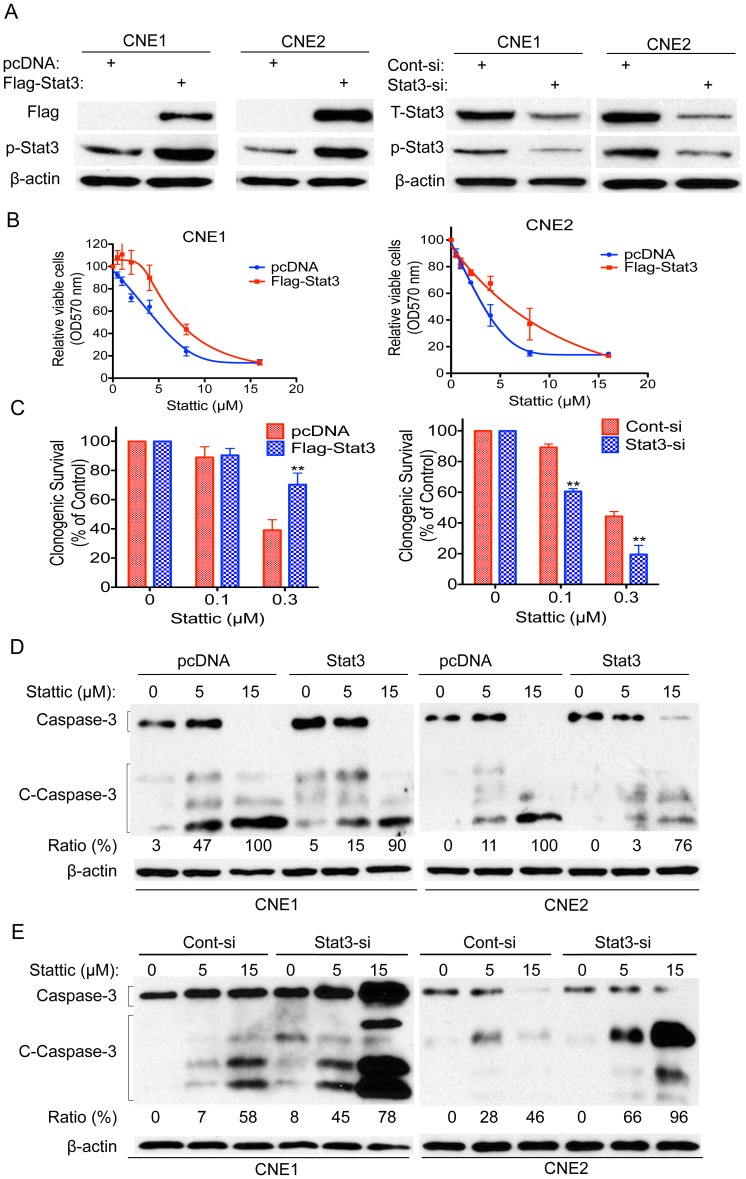
Ectopic Stat3 attenuates Stattic-induced growth inhibition and apoptosis in NPC cells. (A) NPC cells were transfected with pcDNA, Flag-Stat3 plasmids, control siRNA (Cont-si), or Stat3 siRNA (Stat3-si) and checked by western blot for the flag and Stat3 expression. (B) NPC cells described in (A) were treated with Stattic at 0–16 µM and assayed for cell viability by the MTT assay. Cell viability was calculated by the percentage of surviving cells relative to non-treated controls. (C) CNE2 cells were transfected with pcDNA, Flag-Stat3, control siRNA (Cont-si), or Stat3 siRNA (Stat3-si) and then treated with or without Stattic at 0.1 or 0.3 µM for 48 h, followed by examination for colony formation. (D) CNE1 cells (left) and CNE2 cells (right) were transfected with pcDNA or Stat3 plasmids and then exposed to the indicated doses of Stattic for 48 h, and then apoptotic cells were measured by western blot analysis of cleaved caspase-3. (E) CNE1 cells (left) and CNE2 cells (right) were transfected with control siRNA or Stat3 siRNA and then exposed to the indicated doses of Stattic for 48 h, and then apoptotic cells were measured by western blot analysis of cleaved caspase-3. Protein levels were quantified using ImageJ software. DMSO were used as control in “0” groups.

To further explore the role of Stat3 in Stattic action, we sought to determine whether upregulation of Stat3 would influence Stattic efficacy. CNE1 and CNE2 cells were transfected with pcDNA-Stat3 or a control vector. Western blotting showed that ectopic Stat3 and Stat3 siRNA was successfully transfected into NPC cells (Fig. 5A). Forced expression of Stat3 significantly attenuated Stattic-induced growth inhibition. The growth inhibition was decreased by 26% and 19% following Stattic treatment at 4 µM and 8 µM, respectively, in Stat3 plasmid-treated CNE1 cells (Fig. 5B, left). The Stat3 plasmid-treated CNE2 showed decrease sensitivity to Stattic, with decreases of approximately 35% and 10% in the growth inhibition upon treatment with 4 µM and 8 µM Stattic, respectively, compared with the pcDNA-treated controls (Fig. 5B, right). We also tested the effects of ectopic Stat3 on the cells’ response to Stattic using a colony formation assay. We observed results similar to those described above; NPC cells transfected with Stat3 plasmid had better survival rates when exposed to Stattic (Fig. 5C, left). Furthermore, we found that enforced expression of Stat3 attenuated Stattic-induced caspase-3 cleavage in NPC cells (Fig. 5D). Thus, upregulation of Stat3 likely contributes to the decreased sensitivity of the NPC cells to Stattic.

To confirm the above conclusion, we next conducted the reverse experiment; we decreased the Stat3 expression in NPC cells and determined whether it would enhance the sensitivity of NPC cells to Stattic. Thus, NPC cells were transfected with Stat3 siRNA, and cell survival was measured by the colony formation assay. The Stat3 knockdown CNE2 cells displayed increased Stattic-induced cell inhibition, with 29% and 25% higher cell survival inhibition than control cells transfected with a vector at 0.1 and 0.3 µM Stattic treatment, respectively (Fig. 5C, right). Similar results were seen when we tested caspase-3 cleavage. CNE1 cells (Fig. 5E, left) and CNE2 cells (Fig. 5E, right) transfected with Stat3 siRNA displayed increased Stattic-induced caspase-3 cleavage compared with control cells when exposed to Stattic. Considering our findings together, we conclude that Stat3 levels were associated with Stattic efficacy.

### Stattic Enhances the Antitumor Effects of Cisplatin in NPC

**Figure 6:**
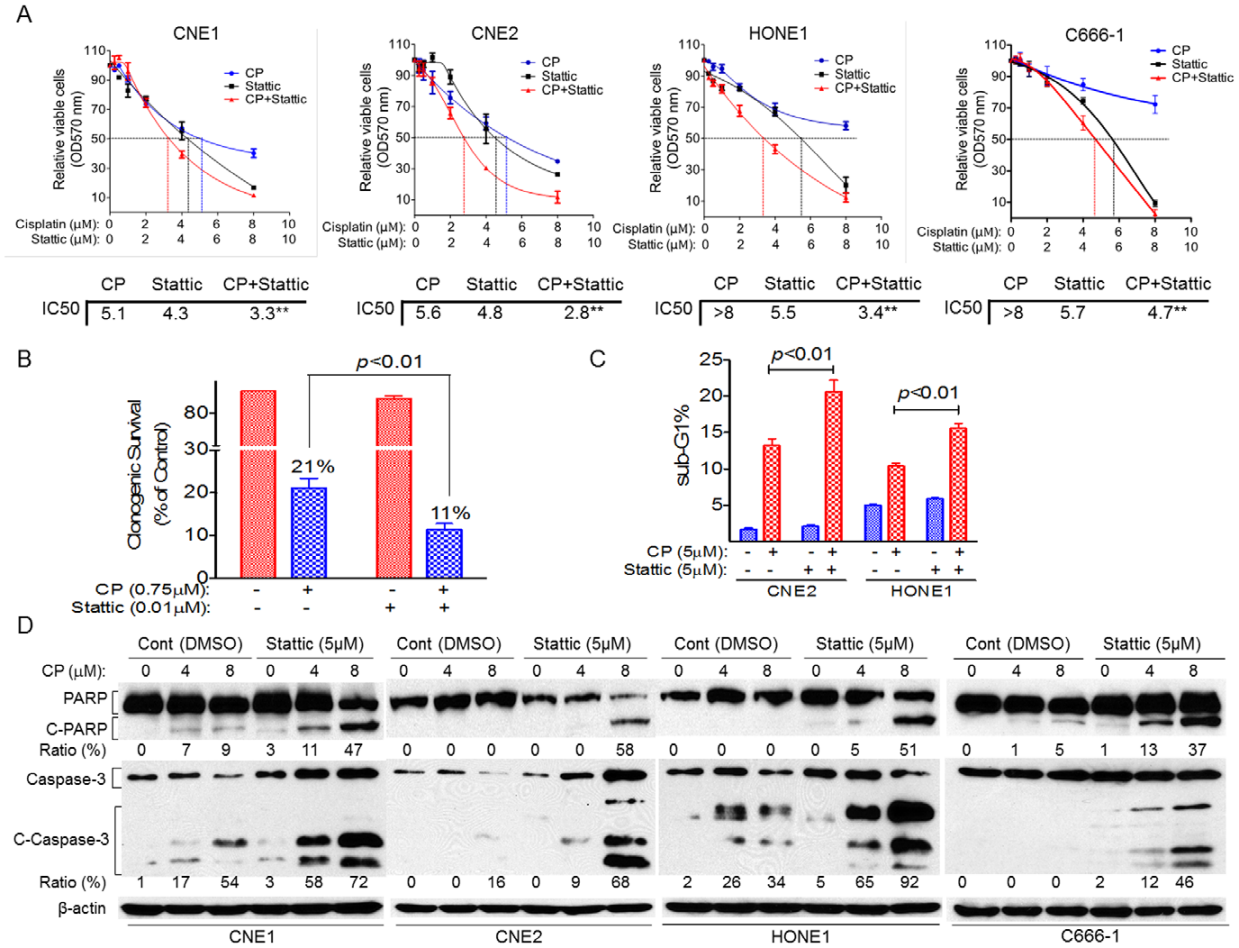
Stattic enhances the antitumor effects of cisplatin in NPC. NPC cells were treated with cisplatin (CP) alone, Stattic alone or both together, 48 h later, cells viability were measured by the MTT assay (A), number of colonies formed were counted in CNE2 cells (B), subG1 cells were analyzed by PI staining (C) and cleaved PARP (C-PARP) and cleaved caspase-3 (C-Caspase 3) was detected by western blot (D). Data are means ± s.d. for three independent experiments. DMSO were used as control in “Cont” groups, PBS were used as control in “0” groups.

Because cisplatin is the main treatment for NPC, we investigated whether Stattic is involved in the antitumor effects of cisplatin. We first used the MTT assay to test whether Stattic enhances the antitumor effects of cisplatin. As shown in Fig. 6A, combined treatment of NPC cells with Stattic and cisplatin for 48 h resulted in enhanced anti-tumor activity of cisplatin. Compared with results for the cisplatin alone treated cells, the IC50 value decreased in combined cisplatin and Stattic treatment group (by 35% in CNE1, 50% in CNE2, more than 57% in HONE1 and more than 41% in C666-1). We also used the colony formation assay to test the effects of Stattic on the cells’ response to cisplatin. We observed results similar to those described above; CNE2 cells treated with Stattic decreased survival rates by 48% when exposed to cisplatin (Fig. 6B).

We further examined whether Stattic could enhance cisplatin-induced apoptosis in NPC cells. We found that cisplatin induced more apoptosis in Stattic-treated cells than in control cells: by 62% increase in CNE2 cells and 57% increase in HONE1 cells, respectively, as measured by PI staining (Fig. 6C). Proteolytic cleavage of PARP and cleaved caspase-3 are the hallmarks of apoptosis. Thus, we also examined the effect of Stattic on the proteolytic cleavage of PARP and cleaved caspase-3 in response to cisplatin. Compared with results for the control cells, cisplatin consistently induced more proteolytic cleavage of PARP (38% change in CNE1 cells, 58% change in CNE2 cells, 51% change in HONE1, and 32% change in C666-1) and cleaved caspase-3 (41% change in CNE1, 52% change in CNE2, 58% change in HONE1, and 44% change in C666-1) in Stattic-treated cells (Fig. 6D).

### Stattic Sensitize NPC Cells to Radiotherapy

**Figure 7:**
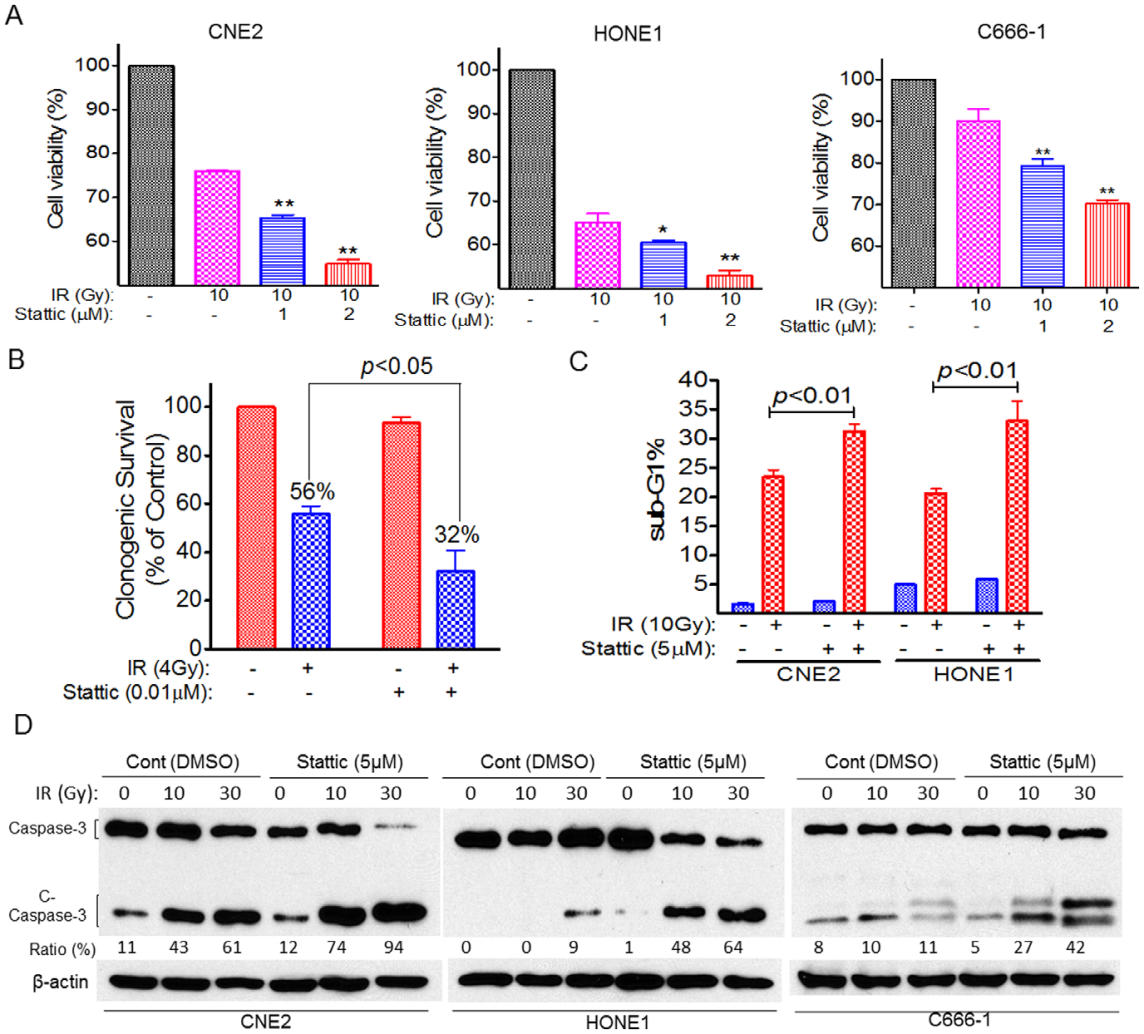
Stattic sensitize NPC to radiotherapy. NPC cells were treated with 10 Gy of IR alone or together with different doses of Stattic for 48 h, followed by detection of cells viability (A), colony formation (CNE2) (B), PI staining (C) and cleaved caspase-3 (C-Caspase 3) (D). DMSO were used as control in “−” groups.

As discussed above, Stattic enhanced the antitumor effect of cisplatin in NPC cells (Fig. 6). We next used a sub-optimal dose (≤IC15) of Stattic to examined whether Stattic increase the sensitivity of NPC cells to IR (10 Gy). As expected, NPC cells with Stattic treatment showed an increase in the efficacy of IR compared with control cells treated with IR alone. The cell viability was reduced by 15% and 28% following Stattic treatment at 1 and 2 µM in CNE2 cells; by 8% and 20% in HONE1 cells; and by 12% and 22% in C666-1 cells (Figure 7A). We also examined the effects of Stattic on cell’s response to IR using a colony formation assay. The survival rates of CNE2 treated with Stattic reduced by 43% when exposed to IR (Figure 7B).

We further examined the influence of Stattic on IR -induced apoptosis in NPC cells. We found that IR induced more apoptosis in Stattic-treated cells than in control cells: by 35% increase in CNE2 cells and 65% increase in HONE1 cells, respectively, as measured by PI staining (Fig. 6C). In addition, NPC cells treated with Stattic displayed increased IR-induced caspase-3 cleavage compared with control cells when exposed to IR (Figures 7D). Compared with results for the control cells, IR consistently induced more proteolytic cleavage of caspase-3 (33% change in CNE2, 55% change in HONE1, and 31% change in C666-1) in Stattic-treated cells (Fig. 7D).

## Discussion

In the current study, we have presented evidence showing the effective inhibition of STAT3 activation by the small molecule inhibitor, Stattic, which resulted in decreased STAT3-mediated cyclin D1 expression and subsequent antitumor effects in NPC cells. These findings suggest that Stattic may be effective in suppressing NPC cell growth in cancer patients with constitutive Stat3 signaling.

Inhibiting the STAT3 signaling pathway may represent an effective strategy in the treatment of NPC, and here we present the first evidence of Stattic activity in NPC. First, we found STAT3 is overexpressed in NPC cell lines but not in paired normal keratinocyte cells; our findings on Stat3 expression also confirm those of previous reports [17]. For instance, Hsiao et al. reported that constitutive activation of STAT3 was detected in 43 (70.5%) of 61 tumor specimens [30]. In addition, Stattic blocked the IL-6-induced Stat3 activation. Our data showed that IL-6 stimulates the growth of NPC cells, a result that is also supported by Tu et al. [27]. Furthermore, our findings showed that Stattic can block IL-6-induced Stat3 activation and cell growth.

Stat3 has become a widely explored target for new drug development [31], [32]. Agents targeting Stat3 include direct inhibitors of Stat3 and the SH2, DNA binding, N-terminal domains, or the upstream mediators of Stat3 activation [33], and a growing body of evidence has shown that the inhibition of constitutively active STAT3 leads to impaired survival and proliferation [13], [34]. Recent studies suggest that treatment with Stattic impaired cell survival and increased radiosensitivity in orthotopic xenograft UM-SCC-17B tumors [35]. However, the potential activity of Stattic on NPC and the radio- and chemo-sensitivity has not been tested.

In this study, we have shown that Stattic is an effective Stat3 inhibitor and had high efficacy against NPC cell viability. Given this finding, we examined the potential effects of Stattic on tumor cell apoptosis. Our results showed that Stattic dramatically induced apoptosis in NPC cells. We also demonstrated that ectopic expression of Stat3 partially abrogates, whereas knockdown of Stat3 enhances, Stattic’s activity against NPC cells. Moreover, We found that Stattic enhanced cisplatin activity in NPC cell lines. A similar therapeutic strategy has been reported in ovarian cancer, in which the combined use of the STAT3 inhibitor S3I-201 circumvented cisplatin resistance [36]. In addition, inhibition of Stat3 function by DPP, another Stat3 inhibitor, resulted in significant decreases in cisplatin resistance and enhanced apoptosis in drug-resistant gastric cancer cells [37]. Furthermore, work with STAT3-targeted shRNA demonstrated enhanced radiosensitivity in the human squamous cell carcinoma cell line A431 [38], and Stattic impaired increased radiosensitivity in orthotopic xenograft UM-SCC-17B tumors. Consistent with these observations, our studies demonstrated that Stattic sensitize the NPC to radiotherapy. By targeting multiple oncogenic signaling pathways, Stattic may be able to sensitize tumors to radiotherapy and chemotherapy. Our finding suggests that a combination of Stattic with cisplatin or radiotherapy may be more effective in treating cancer patients than either drug alone. These results provide supportive evidence that Stattic may be effective in suppressing NPC tumor cell growth in cancer patients with constitutive Stat3 signaling.

In addition to Stattic, several other small molecule inhibitors of STAT3 have been described in the literature, and continuing efforts to develop more potent STAT3 inhibitors are under way [39], [40]. In particular, STA-21 and S3I-201 selectively target the DNA-binding domain of STAT3 and effectively suppress its activity in rhabdomyosarcoma, osteosarcoma, and breast cancer [22], [41]. This new generation of small molecule inhibitors is based on virtual screening of the crystalline structure of STAT3 and has offered promising results. Given the role of STAT3 in modulating tumor viability, radiosensitivity, and chemosensitivity, the development of an efficient STAT3 inhibitor is critical in the development of novel treatment regimens for solid tumors. Our findings emphasize the importance of Stattic in tumor viability and resistance to chemotherapy and radiotherapy. Having demonstrated a valuable therapeutic strategy involving STAT3 inhibition in NPC, this work should provide impetus for the clinical evaluation of biological modifiers that may enhance cisplatin treatment and radiotherapy and potentially reduce undesirable side effects associated with currently available treatment strategies.
