# Correction: Stat3 inhibitor stattic exhibits potent antitumor activity and induces chemo- and radio-sensitivity in nasopharyngeal carcinoma

**DOI:** 10.1371/journal.pone.0237943

**Published:** 2020-08-13

**Authors:** Yunbao Pan, Fuling Zhou, Ronghua Zhang, Francois X. Claret

After publication of this article [1], concerns were raised that the bands in Fig 4C corresponding to PARP from HONE1 NPC cells appear similar to bands in the panel representing PARP from C666-1 NPC cells.

**Fig 4:**
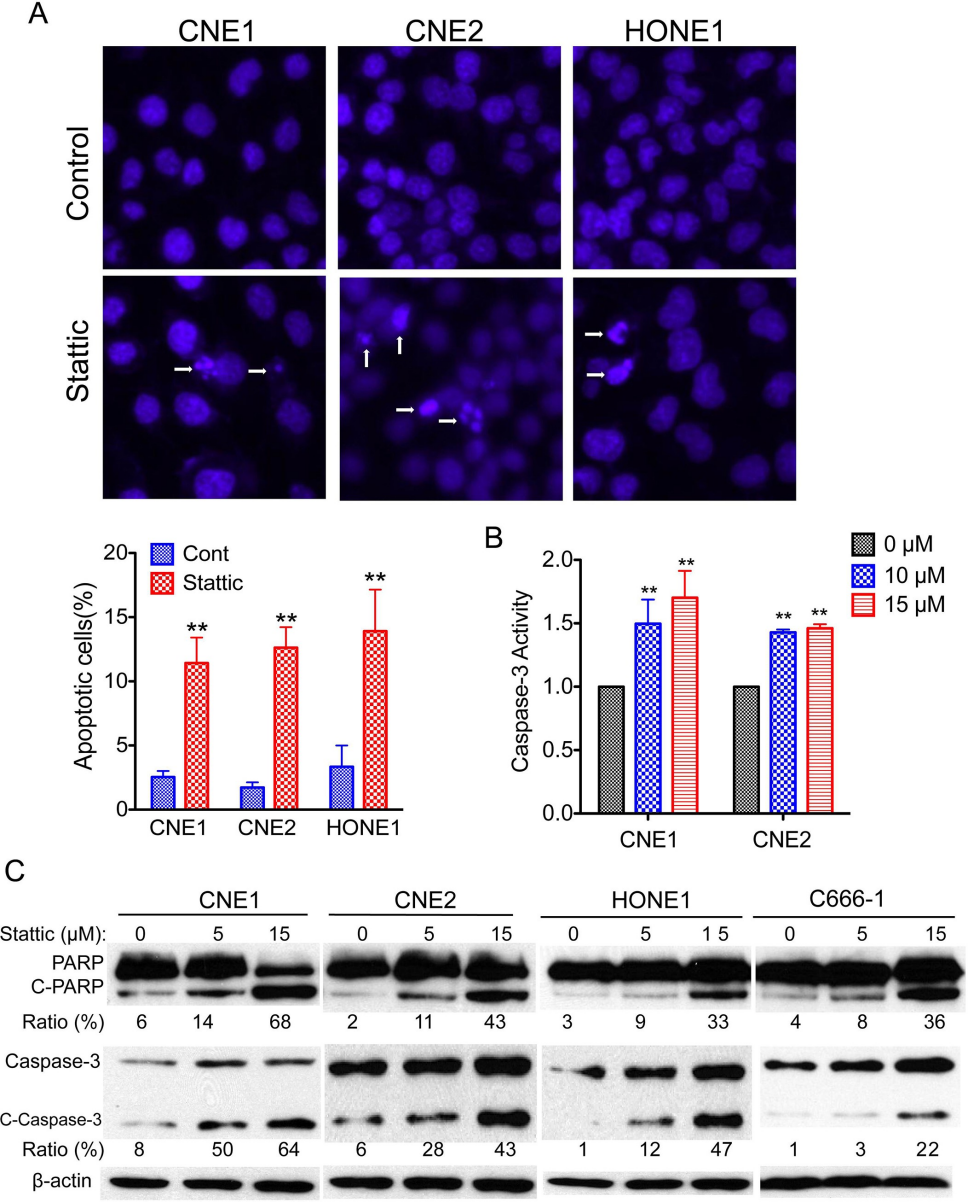
Stattic induces apoptosis in NPC cells. (A) Apoptosis was measured by Hoechst 33342 staining. (Top) NPC cells were treated with 10 μM Stattic for 48 h, nuclei were stained with Hoechst 33342, and imaging analysis was performed as described in the Materials and Methods. The white arrows indicate apoptotic cells. Original magnification, ×200. (Bottom) Quantification of the cell staining. (B) Effect of Stattic on caspase-3 activity. The cells were treated with the indicated concentrations of Stattic for 48 h. The activities were determined as described in Materials and Methods. (C) NPC cells were exposed to the indicated concentrations of Stattic for 48 h; apoptotic cells were measured by western blot analysis of cleaved PARP and cleaved caspase-3. Protein levels were quantified using ImageJ software. Data are means ± s.d. for three independent experiments, *P<0.05, **P<0.01. DMSO were used as control in “0” groups.

The authors clarified that, when preparing the manuscript, the raw data from the C666-1 experiment was mistakenly included in both the C666-1 and HONE1 panels. With this notice, the authors provide an updated Fig 4, along with an original blot image underlying the panels which were originally of concern (in Supporting Information S1 File).

A *PLOS ONE* Editorial Board member reviewed the updated figure and underlying data and confirmed that they support the results and conclusions reported in the original article.

At the time of publication of this notice, the underlying data for the rest of Fig 4 and the other figures in the article are not available.

In addition, concerns have been noted for three of the cell lines used in this study. The CNE-1 and CNE-2 cell lines have been reported as contaminated with HeLa and highly similar to one another [2, 3]. After publication of this article [1], the HONE-1 line was also reported as potentially contaminated with HeLa cells [3]. A further cell line, the Epstein-Barr virus-positive line C666-1, was also used in the study. In light of these issues, there may be limited generalizability of the findings as representative of nasopharyngeal carcinoma biology.

The authors apologize for the errors in the published article. *PLOS ONE* apologizes for our delay in posting this Correction and acknowledges that the authors responded to the concerns about Fig 4 by providing data and clarifications in 2015.

## Supporting information

S1 File Original raw blot images for PARP cleavage region of western blot for HONE1 and C666-1 cells exposed to Stattic, used to generate panels in Fig 4C. (PPTX)

## References

[1] Pan Y, Zhou F, Zhang R, Claret FX (2013) Stat3 Inhibitor Stattic Exhibits Potent Antitumor Activity and Induces Chemo- and Radio-Sensitivity in Nasopharyngeal Carcinoma. PLoS ONE 8(1): e54565 10.1371/journal.pone.0054565 23382914 PMC3558509

[2] Chan SY-Y, Choy K-W, Tsao S-W, Tao Q, Tang T, Chung GT-Y, Lo K-W (2008) Authentication of nasopharyngeal carcinoma tumor lines. Int J Cancer 122(9): 2169–2171. 10.1002/ijc.23374 18196576

[3] Strong MJ, Baddoo M, Nanbo A, Xu M, Puetter A, Lin Z (2014) Comprehensive high-throughput RNA sequencing analysis reveals contamination of multiple nasopharyngeal carcinoma cell lines with HeLa cell genomes. J Virol. 88(18): 10696–704. 10.1128/JVI.01457-14 24991015 PMC4178894

