# Supplementary figures and images for: Correction: Stat3 inhibitor stattic exhibits potent antitumor activity and induces chemo- and radio-sensitivity in nasopharyngeal carcinoma
Source: PLoS One. 2020 Aug 13;15(8):e0237943. doi: 10.1371/journal.pone.0237943 (PMC7425959; doi:10.1371/journal.pone.0237943)

## Slide 1
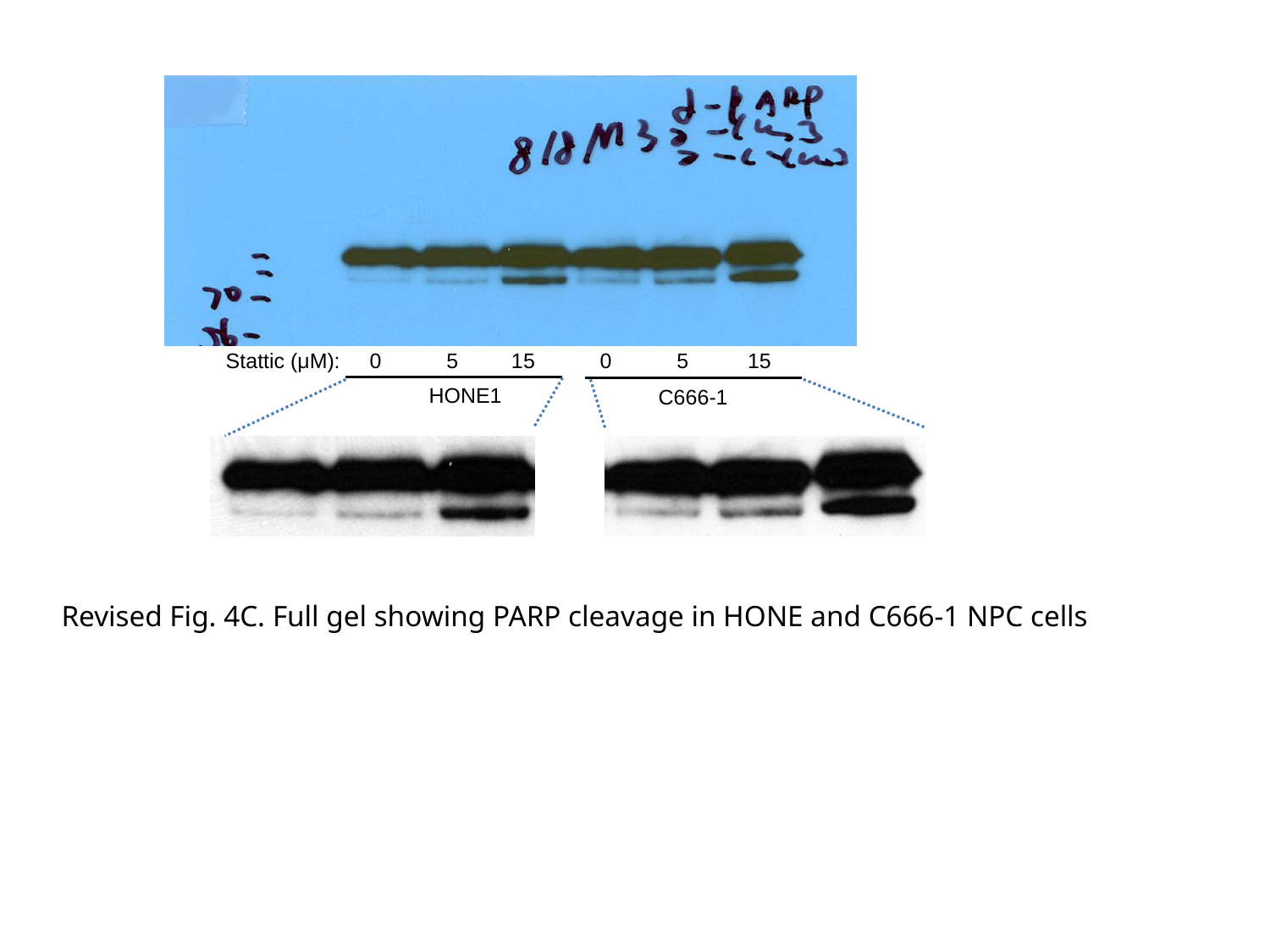

Stattic (μM): 0 5 15 0 5 15
HONE1
C666-1
Revised Fig. 4C. Full gel showing PARP cleavage in HONE and C666-1 NPC cells

Supplement: S1 File — (PPTX) [file pone.0237943.s001.pptx]
